# Exploring the Sociodemographic, Clinical, and Lifestyle Determinants of Hypertension Among Sedentary Workers: A Cross-Sectional Study in the Cape Coast Metropolis

**DOI:** 10.1155/bmri/8818300

**Published:** 2025-08-08

**Authors:** Andrew Oklu, Edmund Addison, Rachel Araba Quaicoe, Addison Bouyena, Frank Naku Ghartey, Richard Armah, Nelson Ekow Kumah, Gabriel Pezahso Kotam, George Nkrumah Osei, Richard K. D. Ephraim

**Affiliations:** ^1^Department of Medical Laboratory Science, School of Allied Health Sciences, University of Cape Coast, Cape Coast, Central Region, Ghana; ^2^Department of Chemical Pathology, School of Medical Sciences, University of Cape Coast, Cape Coast, Central Region, Ghana; ^3^Laboratory Division, Ankaful Psychiatric Hospital, Cape Coast, Central Region, Ghana; ^4^Kidney Research Initiative, Cape Coast, Central Region, Ghana

**Keywords:** Ghana, hypertension, lifestyle, prevalence, sedentary, sociodemographic

## Abstract

**Background:** Hypertension, a major public health concern, is a significant risk factor for a range of cardiovascular diseases and other chronic health conditions, especially in sub-Saharan Africa. This study assessed the prevalence, sociodemographic, lifestyle, and clinical determinants of hypertension among sedentary workers in the Cape Coast Metropolis.

**Methods:** A cross-sectional study was conducted among 170 sedentary workers at Abura Market, Cape Coast. Sociodemographic, lifestyle, and clinical characteristics were assessed. Data was analyzed accordingly using Statistical Package for Social Sciences (SPSS) Version 26 (IBM Inco., United States).

**Results:** The study involved 170 participants, of which 127 were females [median age = 42]. The prevalence of hypertension was found to be 18.2% (31/170). Increased consumption of alcohol, engagement in physical activity, fruit intake, weight, body mass index, waist circumference, hip circumference, and waist-to-hip ratio were observed among hypertensive participants compared to those without hypertension. An increased risk of hypertension incidence was found to be significantly associated with age (*p* < 0.001), having a family history of hypertension (*p* < 0.001), and experiencing shortness of breath (*p* = 0.011) in a multivariate analysis. Having a family history of hypertension and experiencing shortness of breath increase the odds of an individual developing hypertension by 23.9 and 9.49-fold.

**Conclusion:** The study reveals a higher prevalence of hypertension among sedentary workers. High-risk groups for hypertension include the aged, those with a family history of hypertension, and those with diabetes mellitus. Given the lack of statistically significant symptoms, hypertension may remain undetected in individuals. The findings therefore underscore the need for public health interventions.

## 1. Introduction

Hypertension, a global health concern, is a chronic condition characterized by consistently elevated blood pressure levels [1–4]. Hypertension prevalence worldwide is increasing due to aging populations adopting sedentary lifestyles [5]. It is a significant risk factor for a range of cardiovascular diseases, stroke, kidney disorders, other chronic health conditions, and death, especially in sub-Saharan Africa [1, 2, 6–15]. The first national study in Ghana revealed a hypertension prevalence of 13.1% among individuals aged 15–49 years, with a 23.0% and 52.5% prevalence for hypertension and prehypertension, respectively, reported among taxi drivers in the Cape Coast metropolis [15, 16]. Studies by Opoku et al. [13] also found the prevalence of hypertension to be 30.43% and 11.48% as per the American College of Cardiology/American Heart Association and Joint National Committee 7 guidelines, respectively, in Ghana [13]. This underscores the need for targeted public health interventions and healthcare initiatives to address and manage this condition.

Several lifestyle and environmental factors, particularly social and economic factors, contribute to the incidence and progression of hypertension in different groups of people [5, 17–19]. Sociodemographic factors such as an increase in age, low educational attainment, and low socioeconomic status have been identified as crucial determinants of hypertension risk [8, 10, 20, 21]. Sedentary activities such as taxi driving, trading, and shopkeeping predispose individuals to developing cardiovascular disease, cancer, metabolic disorders such as hypertension, diabetes mellitus, obesity, and dyslipidemia [22–29]. This is due to the fact that a sedentary lifestyle decreases physical activities, increases weight gain and adiposity, and subsequently decreases cardiac output and systemic blood flow [28–31].

Despite the increased risk and incidence of hypertension among occupations involving prolonged sitting or minimal physical activity, limited studies have been conducted to assess specific sociodemographic, lifestyle, and clinical determinants that contribute to this disease burden. This study assessed the prevalence, sociodemographic, lifestyle, and clinical determinants of hypertension among sedentary workers in the Cape Coast Metropolis. The findings of this study would aid in disease burden estimation and also facilitate targeted preventive measures and early intervention to reduce the incidence and progression of hypertension.

## 2. Methodology

### 2.1. Study Site, Design, Duration, and Sample Size

A cross-sectional study was conducted among sedentary workers from May 17 to May 18, 2023, at the Abura Market, Cape Coast. Abura Market, a major commercial center in Cape Coast Metropolis, comprises a sedentary workforce including vendors, salespersons, traders, and shopkeepers, providing an accessible group for this study. The disproportionately high number of female participants in our study reflects the composition of the Abura Market, the data collection site primarily comprising women as the main traders and vendors. A nonrandomized convenience sampling approach was used to recruit 170 participants.

### 2.2. Eligibility Criteria

Sedentary workers, including salespersons, vendors, traders, shopkeepers, and all individuals whose activities revolved around sitting or standing for 6 h or more with minimal physical activity, were eligible for the study. Sedentariness was assessed using a domain-based approach, focusing on time spent sitting or standing at work with minimal physical activity. Those reporting ≥ 6 h/day of such low-activity behavior were considered sedentary [32]. Physical activity was estimated through participants' self-reported frequency and duration of domestic tasks such as cleaning, laundry, carrying loads, and other light to moderate activities which served as proxies for overall activity levels. Individuals below 18 years of age and those whose work involved moderate to high physical activities were excluded from the study. Exclusion was determined through initial screening questions that assessed age, occupational demands, and estimated daily activity levels.

### 2.3. Data Collection

#### 2.3.1. Collection of Sociodemographic, Clinical, and Therapeutic Data

Sociodemographic data (age, gender, religious affiliation, level of education, marital status, occupation, ethnicity, and place of residence), lifestyle data (smoking, alcohol intake, physical activities, fruit consumption), and clinical data (diagnosis and duration of hypertension and diabetes mellitus status, symptoms and family history of hypertension) were obtained from the participants using a pretested interviewer-administered questionnaire adapted from the WHO STEPS instrument for surveillance of chronic disease risk [33]. The adapted questionnaire collected information on sociodemographic factors, lifestyle, and clinical details through a combination of close-ended questions with predefined responses. Physical activity was measured based on self-reported frequency and duration of mild, moderate, and vigorous activities undertaken in a typical week. Cultural and lingual modifications were made to suit the specific objectives and the population demographics.

#### 2.3.2. Measurement of Blood Pressure, Weight, Height, and Waist and Hip Circumference and Estimation of Body Mass Index (BMI) and Waist-to-Hip Ratio

After collecting demographics, clinical, and therapeutic data, participants were led to a quiet place to sit and relax for at least 10 min at the survey site before measuring blood pressure and anthropometric data. Blood pressure in the right arm in the seated position was measured using a mercury sphygmomanometer (Omron blood pressure monitor, model BP742N) and a stethoscope and recorded three times at 1-min intervals. If the mean systolic blood pressure (SBP) was ≥ 140 mmHg and/or the mean diastolic blood pressure (DBP) was ≥ 90 mmHg, or if the subject reported having been diagnosed with hypertension and taking antihypertensive medications, he or she was considered a case of hypertension. This is in accordance with the Seventh Report of the Joint National Committee on Prevention, Detection, Evaluation, and Treatment of High Blood Pressure [34]. All blood pressure measurements were done following recommendations of the American Heart Association standards [35].

Using a wall-mounted graduated ruler (BAHCO, United Kingdom) and a weighing balance (Seca GmbH & Co. KG, Germany), the height and weight (to the nearest 0.1 kg) in light clothing of participants were measured. The BMI was then calculated as the ratio of the weight (kilogram) and the square of the height (square meter) [36]. Waist circumference (centimeter) and hip circumference (centimeter) were taken with a tape measure as the point midway between the costal margin and iliac crest in the midaxillary line and at the widest point around the greater trochanter, respectively, with participants standing and breathing normally [37, 38]. The waist-to-hip ratio was calculated as the waist circumference divided by the hip circumference [37, 38].

### 2.4. Data Analysis

Initial entry and organization of data were done using Microsoft Excel (Version 2013). The data were cleaned and imported into the Statistical Package for Social Sciences (SPSS) Version 26 (IBM Inco., United States) for analysis. Qualitative variables were presented as frequencies and percentages. Quantitative variables were evaluated for normality using skewness, kurtosis, and both the Kolmogorov–Smirnov and Shapiro–Wilk tests. Since the data were not normally distributed, values are reported as medians with interquartile ranges. Also, correlational analysis, chi-square test, and Fisher's exact test were used to analyze associations between categorical variables. The presence of hypertension was considered the dependent variable. Evaluation of the correlation between the dependent and independent variables was performed using the Wilcoxon rank sum test. All analyses were done at a 95% confidence interval, and *p* values less than or equal to 0.05 were considered statistically significant.

## 3. Results

The study involved 170 participants, with most being females (127/170) [median age = 42] (Table 1). The majority of the participants were Christians (81.0%), married (56.0%), employed (75.0%), Akan (77.0%), had tertiary education (55%), and lived in a periurban location (55%) (Table 1).

The majority of the participants had no current alcohol consumption (81.0%) or previous alcohol consumption (80.0%), did not smoke (100%), engaged in physical activities (85.0%), and consumed fruits (94.0%) (Table 2). Consumption of alcohol (32%), engagement in physical activity (90%), and fruit intake (100%) were found to be higher among hypertensive participants compared to nonhypertensive ones (Table 2).

Table 3 shows the clinical characteristics of study participants. Increased weight, BMI, waist circumference, hip circumference, waist-to-hip ratio, and systolic and diastolic pressure were observed among hypertensive participants compared to those without hypertension (Table 3).

The prevalence of hypertension was found to be 18.2% (31/170) with female predominance (25/31) (Table 4). Compared to 77% of participants with hypertension, only 28% of nonhypertensive participants had a family history of hypertension (Table 4). Having a family history of hypertension (*p* < 0.001), having shortness of breath (*p* = 0.011), experiencing confusion (*p* = 0.003), anxiety (*p* = 0.021), and abnormal heart rhythm (*p* < 0.001) showed statistically significant differences between hypertensive and nonhypertensive participants (Table 4).

Age, having a family history of hypertension, and experiencing shortness of breath were significantly associated with increased risk of hypertension incidence in the multivariate analysis (Table 5). The multivariate analysis also shows that for every increase in age by 1 (year), the odds of a person getting hypertension increase by 1.14-fold. Also, having a family history of hypertension and having shortness of breath increase the odds of an individual developing hypertension by 23.9 and 9.49-fold (Table 5).

## 4. Discussion

Sedentary behavior, a significant public health concern, is associated with an increased risk of cardiovascular diseases, obesity, and adverse metabolic profiles [24]. This study assessed the prevalence, sociodemographic, lifestyle, and clinical determinants of hypertension among sedentary workers in the Cape Coast Metropolis.

The prevalence of hypertension in our study was found to be 18.2%. This prevalence is higher than that observed in similar studies by Nyarko [39] (7.5%), Sanuade et al. [15] (13.0%), and Oyekale [40] (13.28%) in Ghana [15, 39, 40]. The variations observed may be attributed to the study participants involved in various studies. Nyarko, Sanuade et al., and Oyekale employed women in the reproductive age, persons aged 15–49 years, and women aged 15–49 years, respectively, while our study used both sexes and persons with a sedentary lifestyle. However, the prevalence found in our study is lower than the findings of Setorglo et al., Addo et al., and Odugbemi et al. among taxi drivers (23%), civil servants (30.3%), and traders (34.8%) in Ghana and Nigeria but projects an increased risk of hypertension incidence among individuals with sedentary behavior, as observed by Laxmaiah et al., who found the risk of hypertension to be 1.39 times higher among sedentary workers compared to moderate or heavy workers [11, 16, 22, 28, 41, 42]. This high prevalence of hypertension among individuals with less physical activity may be due to various mechanisms, including alteration in the cardiac output and total peripheral vascular resistance, as well as reduction in the metabolic demands and systemic blood flow [28].

In agreement with studies by Amugsi et al., Eghabli et al., Laxmaiah et al., Choi et al., Bosu et al., and Ghorbani et al. in Iran, India, West Africa, and Korea which observed hypertension prevalence to significantly increase with age, our study found age to be a significant risk factor for hypertension incidence which increases by 1.14-fold for every increase in age by 1 year [1, 9, 11, 43–45]. This finding may be attributed to the changes that take place in the vascular walls as one ages, such as arterial stiffening, which in turn affects blood pressure levels [46]. This indicates age as a major risk factor for hypertension prevalence and hence should be considered when developing strategies for the prevention and control of hypertension.

Increased weight, BMI, waist circumference, hip circumference, and waist-to-hip ratio were observed to increase among hypertensive participants compared to those without hypertension. Similar findings were also observed in studies by Amugsi et al. [1], Anwar et al. [47], Bramlage et al. [48], Dosoo et al. [2], Konlan et al. [10], Kumar et al. [3], Laxmaiah et al. [11], Mohammed et al. [49], and Oyekale [40] [1–3, 10, 11, 40, 47–49]. Other similar studies also found a higher prevalence of hypertension, ranging from 60% to 77%, among obese and overweight individuals than normal-weight subjects [48, 50]. This may be attributed mostly to the deposition of adipose tissues in the perivascular and vascular spaces, leading to the formation of plaques and thickening of the vessels [50]. Improvements in blood pressure levels have, however, been highlighted by some studies to result from decreased body weight, BMI, and waist circumference [50–53]. This therefore emphasizes the importance of interventions to reduce weight, BMI, waist circumference, hip circumference, and waist-to-hip ratio to help control and prevent high blood pressure levels [1, 47].

The majority of hypertensive participants reported having severe headaches but experienced no chest pain, shortness of breath, dizziness, vomiting, blurred vision, confusion, and anxiety. Similar studies have also reported an increased prevalence of no symptoms and unawareness of disease among hypertensive participants, especially among market women, including traders [9, 43, 54]. This may contribute to the widespread prevalence and neglect of this condition, especially among persons with a sedentary lifestyle. Individuals at risk of hypertension, including sedentary workers and the aged, should therefore undergo regular check-ups to know their hypertensive status for early detection and control.

In consonance with our study which found having a family history of hypertension (77%), no diabetes mellitus (84%), and no smoking (100%) to be prevalent among hypertensive participants, Eghbali et al. also found increased prevalence of the same parameters among hypertensive patients (having family history of hypertension 277 [76.1%], no diabetes mellitus 266 [73.1%], and no smoking [90.1%]) [9]. This finding, however, differed from studies by Li et al. and Apidechkul et al. in China and Thailand, respectively, which found smoking and not exercising (60.8%) to be more prevalent among hypertensives [17, 55]. This variation may be attributed to the study populations and sample sizes employed in various studies, as Li et al. and Apidechkul et al. employed the general population and not a specific group of people (sample size = 10,648 and 2552, respectively). Also, having a family history of hypertension and being diagnosed with diabetes mellitus increases the odds of an individual developing hypertension by 23.9 and 0.11-fold. These findings align with conclusions drawn from similar studies [2, 10]. It is therefore important to pay more attention to individuals who fall within this high-risk group to facilitate early intervention and targeted preventive measures.

Interestingly, our study found that reported physical activity and fruit intake were higher among hypertensive participants, a finding that contradicts conventional understanding and prior studies. While unexpected, several plausible explanations may account for this trend. First, this may reflect reverse causation, where individuals who have already been diagnosed or regularly monitor their blood pressure may have subsequently adopted healthier lifestyles as part of their disease management. Additionally, it is possible that the self-reported nature of these lifestyle behaviors may have introduced recall or social desirability bias.

Our study had a few limitations. To begin with, our sample size was small, and the majority of the participants were women; therefore, the results may not be representative of the general population of Ghana. Also, there may be a possibility of underestimated hypertension prevalence among the population since blood pressure measurements were collected only for the 2-day visit.

## 5. Conclusion

The findings of this study reveal a substantial burden of hypertension among individuals with sedentary occupations in the Cape Coast Metropolis. The aged, individuals with a family history of hypertension, and those with diabetes mellitus were found to be high-risk groups for hypertension. The findings of this study therefore emphasize the need for public health interventions for hypertension control among sedentary workers, especially those with a family history of hypertension, diabetes mellitus diagnosis, and the aged.

## Figures and Tables

**Table 1:** Sociodemographic characteristics of participants.

**Characteristics**	**Overall, ** **N** = 170^**a**^	**F, ** **N** = 127^**a**^	**M, ** **N** = 43^**a**^	**p** ** value** ^**b**^	**NH, ** **N** = 139^**a**^	**H, ** **N** = 31^**a**^	**p** ** value** ^**b**^
Age	42 (33, 53)	42 (35, 54)	37 (25, 50)	0.10	38 (31, 50)	55 (48, 63)	**< 0.001**
Gender							0.40
Female					102 (73.0%)	25 (81.0%)	
Male					37 (27.0%)	6 (19.0%)	
Religious affiliation				0.14			> 0.99
Christianity	137 (81.0%)	99 (78.0%)	38 (88.0%)		112 (81.0%)	25 (81.0%)	
Islamic	33 (19.0%)	28 (22.0%)	5 (12.0%)		27 (19.0%)	6 (19.0%)	
Level of education				**< 0.001**			**0.008**
None	17 (10.0%)	17 (13.0%)	0 (0.0%)		10 (7.2%)	7 (22.6%)	
Primary	13 (7.0%)	12 (10.0%)	1 (2.0%)		12 (8.6%)	1 (3.2%)	
SHS	47 (28.0%)	32 (25.0%)	15 (35.0%)		44 (31.7%)	3 (9.7%)	
Tertiary	93 (55.0%)	66 (52.0%)	27 (63.0%)		73 (52.5%)	20 (64.5%)	
Marital status				**0.008**			**0.002**
Divorced/widowed	26 (15.0%)	25 (20.0%)	1 (2.3%)		18 (13.0%)	8 (25.8%)	
Married	95 (56.0%)	71 (56.0%)	24 (55.8%)		74 (53.0%)	21 (67.7%)	
Single	49 (29.0%)	31 (24.0%)	18 (41.9%)		47 (34.0%)	2 (6.5%)	
Occupation				0.88			0.44
Employed	128 (75.0%)	96 (76.0%)	32 (74.0%)		103 (74.0%)	25 (81.0%)	
Unemployed	42 (25.0%)	31 (24.0%)	11 (26.0%)		36 (26.0%)	6 (19.0%)	
Ethnicity				0.10			0.22
Akan	131 (77.0%)	102 (80.3%)	29 (67.0%)		105 (75.5%)	26 (83.9%)	
Ewe	10 (5.9%)	6 (4.7%)	4 (9.3%)		9 (6.5%)	1 (3.2%)	
Ga-Adangbe	8 (4.7%)	3 (2.4%)	5 (12.0%)		5 (3.6%)	3 (9.7%)	
Mole-Dagbani	9 (5.3%)	7 (5.5%)	2 (4.7%)		8 (5.8%)	1 (3.2%)	
Other	12 (7.1%)	9 (7.1%)	3 (7.0%)		12 (8.6%)	0 (0%)	
Place of residence				**< 0.001**			0.20
Periurban	93 (55.0%)	80 (63.0%)	13 (30.2%)		72 (52.0%)	21 (68.0%)	
Rural	21 (12.0%)	19 (15.0%)	2 (4.7%)		17 (12.0%)	4 (13.0%)	
Urban	56 (33.0%)	28 (22.0%)	28 (65.1%)		50 (36.0%)	6 (19.0%)	

*Note:* Entries in bold imply statistical significance.

Abbreviations: H, hypertensive participants; NH, nonhypertensive participants.

^a^Median (IQR); *n* (%).

^b^Wilcoxon rank sum test; Pearson's chi-squared test; Fisher's exact test.

**Table 2:** Lifestyle characteristics of participants.

**Characteristic**	**Overall, ** **N** = 170^**a**^	**F, ** **N** = 127^**a**^	**M, ** **N** = 43^**a**^	**p** ** value** ^**b**^	**NH, ** **N** = 139^**a**^	**H, ** **N** = 31^**a**^	**p** ** value** ^**b**^
Do you smoke							
No	170 (100%)	127 (100%)	43 (100%)		139 (100%)	31 (100%)	
Alcohol consumption				**0.003**			**0.046**
No	137 (81%)	109 (86%)	28 (65%)		116 (83%)	21 (68%)	
Yes	33 (19%)	18 (14%)	15 (35%)		23 (17%)	10 (32%)	
Previous alcohol consumption				0.95			0.87
No	127 (80%)	99 (80%)	28 (80%)		105 (80%)	22 (81%)	
Yes	31 (20%)	24 (20%)	7 (20%)		26 (20%)	5 (19%)	
Physical activity				0.84			0.42
No	26 (15%)	19 (15%)	7 (16%)		23 (17%)	3 (9.7%)	
Yes	144 (85%)	108 (85%)	36 (84%)		116 (83%)	28 (90.3%)	
Intensity of physical activity				0.50			0.52
Mild	51 (35.7%)	37 (34.6%)	14 (38.9%)		44 (38%)	7 (26%)	
Moderate	90 (62.9%)	69 (64.5%)	21 (58.3%)		70 (60.3%)	20 (74%)	
Vigorous	2 (1.4%)	1 (0.9%)	1 (2.8%)		2 (1.7%)	0 (0%)	
Fruit consumption				0.47			0.22
No	11 (6.5%)	7 (5.5%)	4 (9.3%)		11 (7.9%)	0 (0%)	
Yes	159 (93.5%)	120 (93.5%)	39 (90.7%)		128 (92.1%)	31 (100%)	

*Note:* Entries in bold imply statistical significance.

Abbreviations: H, hypertensive participants; NH, nonhypertensive participants.

^a^ *n* (%).

^b^Pearson's chi-squared test; Fisher's exact test.

**Table 3:** Clinical characteristics of study participants.

**Characteristic**	**Overall, ** **N** = 170^**a**^	**F, ** **N** = 127^**a**^	**M, ** **N** = 43^**a**^	**p** ** value** ^**b**^	**NH, ** **N** = 139^**a**^	**H, ** **N** = 31^**a**^	**p** ** value** ^**b**^
Weight	72 (62, 82)	71 (62, 82)	76 (64, 83)	0.50	71 (61, 82)	75 (65, 85)	0.19
Height	2 (2, 5)	2 (2, 5)	2 (2, 6)	**< 0.001**	2 (2, 5)	2 (2, 5)	0.22
BMI	24 (3, 31)	27 (3, 32)	21 (2, 26)	**< 0.001**	23 (3, 30)	27 (3, 33)	0.092
Waist circumference	37 (33, 42)	37 (33, 42)	38 (32, 88)	0.48	37 (32, 43)	40 (36, 42)	0.25
Hip circumference	43 (39, 47)	43 (39, 47)	41 (39, 97)	0.57	43 (39, 47)	44 (40, 47)	0.63
Waist-to-hip ratio	0.88 (0.82, 0.92)	0.87 (0.81, 0.91)	0.89 (0.83, 0.93)	0.15	0.87 (0.82, 0.91)	0.91 (0.86, 0.94)	**0.025**
Systolic pressure	126 (112, 141)	126 (112, 142)	126 (112, 135)	0.89	124 (110, 138)	137 (128, 148)	**< 0.001**
Diastolic pressure	78 (70, 85)	78 (69, 84)	78 (71, 85)	0.76	76 (68, 84)	80 (77, 90)	**0.003**
Pulse	79 (72, 86)	80 (73, 88)	76 (70, 80)	**0.003**	79 (72, 87)	78 (72, 84)	0.34

*Note:* Entries in bold imply statistical significance.

Abbreviations: H, hypertensive participants; NH, nonhypertensive participants.

^a^Median (IQR).

^b^Wilcoxon rank sum test; BMI, body mass ratio.

**Table 4:** Clinical history and symptoms of hypertension among study participants.

**Characteristic**	**Overall, ** **N** = 170^**a**^	**F, ** **N** = 127^**a**^	**M, ** **N** = 43^**a**^	**p** ** value** ^**b**^	**NH, ** **N** = 139^**a**^	**H, ** **N** = 31^**a**^	**p** ** value** ^**b**^
Diagnosis of hypertension				0.40			
No	139 (82%)	102 (80%)	37 (86%)				
Yes	31 (18%)	25 (20%)	6 (14%)				
Family history of hypertension				0.17			**< 0.001**
No	105 (62.5%)	75 (60%)	30 (71%)		98 (72%)	7 (23%)	
Yes	63 (37.5%)	51 (40%)	12 (29%)		39 (28%)	24 (77%)	
Diagnosis of diabetes mellitus				0.11			0.14
No	155 (91.7%)	119 (93.7%)	36 (86%)		129 (93.5%)	26 (84%)	
Yes	14 (8.3%)	8 (6.3%)	6 (14%)		9 (6.5%)	5 (16%)	
Severe headaches				**< 0.001**			0.080
At times	1 (0.6%)	0 (0%)	1 (2.3%)		1 (0.7%)	0 (0%)	
No	83 (48.8%)	53 (42%)	30 (69.8%)		73 (52.5%)	10 (32%)	
Yes	86 (50.5%)	74 (58%)	12 (27.9%)		65 (46.8%)	21 (68%)	
Chest pain				0.73			0.24
No	119 (70%)	88 (69%)	31 (72%)		100 (72%)	19 (61%)	
Yes	51 (30%)	39 (31%)	12 (28%)		39 (28%)	12 (39%)	
Shortness of breath				0.074			**0.011**
No	132 (77.6%)	96 (76%)	36 (83.7%)		114 (82%)	18 (58%)	
Not often	1 (0.6%)	0 (0%)	1 (2.3%)		1 (0.7%)	0 (0%)	
Yes	37 (21.8%)	31 (24%)	6 (14%)		24 (17.3%)	13 (42%)	
Dizziness				**0.040**			0.60
At times	1 (0.6%)	0 (0%)	1 (2.3%)		1 (0.7%)	0 (0%)	
No	123 (72.4%)	88 (69%)	35 (81.4%)		102 (73.4%)	21 (68%)	
Yes	46 (27%)	39 (31%)	7 (16.3%)		36 (25.9%)	10 (32%)	
Vomiting				0.64			> 0.99
No	164 (96.5%)	123 (96.9%)	41 (95.3%)		134 (96.4%)	30 (96.8%)	
Yes	6 (3.5%)	4 (3.1%)	2 (4.7%)		5 (3.6%)	1 (3.2%)	
Blurred vision				**0.002**			0.24
No	114 (67%)	77 (61%)	37 (86%)		96 (69%)	18 (58%)	
Yes	56 (33%)	50 (39%)	6 (14%)		43 (31%)	13 (42%)	
Confusion				**0.017**			**0.003**
No	137 (81%)	97 (76%)	40 (93%)		118 (85%)	19 (61%)	
Yes	33 (19%)	30 (24%)	3 (7.0%)		21 (15%)	12 (39%)	
Anxiety				0.95			**0.021**
No	131 (77%)	98 (77%)	33 (77%)		112 (81%)	19 (61%)	
Yes	39 (23%)	29 (23%)	10 (23%)		27 (19%)	12 (39%)	
Abnormal heart rhythm				**0.002**			**< 0.001**
No	114 (67%)	77 (61%)	37 (86%)		101 (73%)	13 (42%)	
Yes	56 (33%)	50 (39%)	6 (14%)		38 (27%)	18 (58%)	

*Note:* Entries in bold imply statistical significance.

Abbreviations: H, hypertensive participants; NH, nonhypertensive participants.

^a^ *n* (%).

^b^Pearson's chi-squared test; Fisher's exact test.

**Table 5:** Factors independently associated with increased risk of hypertension among participants.

**Characteristic**	**Univariate**				**Multivariable**		
	**N**	**OR** ^**a**^	**95% CI** ^**a**^	**p** ** value**	**OR** ^**a**^	**95% CI** ^**a**^	**p** ** value**
Age	170	1.10	1.06, 1.15	**< 0.001**	1.14	1.06, 1.24	**0.002**
Gender	170						
Female		—	—		—	—	
Male		0.66	0.23, 1.65	0.40	2.77	0.40, 21.3	0.3
Level of education	170						
None		1.94	0.60, 6.17	0.26	0.97	0.09, 8.80	> 0.9
Primary		0.23	0.01, 1.36	0.18	0.19	0.00, 3.77	0.3
SHS		0.19	0.04, 0.64	**0.014**	0.22	0.02, 1.91	0.2
Tertiary		0.52	0.18, 1.43	0.22	0.63	0.06, 6.26	0.7
Marital status	170						
Divorced/widowed		—	—		—	—	
Married		0.64	0.25, 1.74	0.36	1.38	0.14, 13.9	0.8
Single		0.10	0.01, 0.43	**0.005**	0.50	0.01, 17.5	0.7
Ethnicity	170						
Akan		—	—		—	—	
Ewe		0.45	0.02, 2.55	0.46	2.67	0.08, 55.0	0.5
Ga-Adangbe		2.42	0.47, 10.5	0.25	1.75	0.14, 25.2	0.7
Mole-Dagbani		0.50	0.03, 2.93	0.53	1.32	0.04, 23.8	0.9
Other		0.00		0.99	0.00		> 0.9
Location	170						
Periurban		—	—		—	—	
Rural		0.81	0.21, 2.47	0.72	0.83	0.05, 9.93	0.9
Urban		0.41	0.14, 1.04	0.075	1.36	0.15, 12.9	0.8
Alcohol consumption	170						
No		—	—		—	—	
Yes		2.40	0.97, 5.70	**0.050**	0.46	0.06, 3.00	0.4
BMI	170	1.03	1.00, 1.06	0.080	1.01	0.97, 1.06	0.6
Waist-to-hip ratio	170	33.5	0.17, 9771	0.21	57.1	0.00, 580,437	0.4
Family history of hypertension	168						
No		—	—		—	—	
Yes		8.62	3.59, 23.2	**< 0.001**	23.9	4.88, 174	**< 0.001**
Diagnosis of diabetes mellitus	169						
No		—	—		—	—	
Yes		2.76	0.79, 8.68	0.090	0.11	0.01, 0.87	**0.046**
Systolic pressure	169	1.04	1.02, 1.07	**< 0.001**	1.01	0.95, 1.08	0.7
Diastolic pressure	169	1.05	1.02, 1.08	**0.004**	1.07	0.99, 1.18	0.12
Severe headaches	170						
At times		—	—		—	—	
No		788,741	0.00, NA	> 0.99	79,369	0.00, NA	> 0.9
Yes		1,860,216	0.00, NA	> 0.99	626,388	0.00, NA	> 0.9
Shortness of breath	170						
No		—	—		—	—	
Not often		0.00		> 0.99	0.00		> 0.9
Yes		3.43	1.47, 7.94	**0.004**	9.49	1.46, 86.5	**0.027**
Confusion	170						
No		—	—		—	—	
Yes		3.55	1.48, 8.37	**0.004**	2.50	0.42, 16.6	0.3
Anxiety	170						
No		—	—		—	—	
Yes		2.62	1.12, 6.02	**0.024**	1.69	0.29, 9.97	0.6
Abnormal heart rhythm	170						
No		—	—		—	—	
Yes		3.68	1.66, 8.39	**0.002**	0.60	0.11, 2.91	0.5

*Note:* Entries in bold imply statistical significance.

^a^OR = odds ratio, CI = confidence interval.

## Data Availability

Data are available on request from authors.
